# Physical activity and associated factors from a cross-sectional survey among adults in northern Tanzania

**DOI:** 10.1186/s12889-017-4512-4

**Published:** 2017-06-20

**Authors:** Beatrice John, Jim Todd, Innocent Mboya, Mary Mosha, Mark Urassa, Tara Mtuy

**Affiliations:** 1 Department of Epidemiology and Biostatistics, Institute of Public Health, Kilimanjaro Christian Medical University College, P.O. Box 2240, Moshi, Tanzania; 2 Department of Community Medicine, Institute of Public Health, Kilimanjaro Christian Medical University College, P.O. Box 2240, Moshi, Tanzania; 3 London School of Hygiene and Tropical Medicine, Keppel Street, London, UK; 4 National Institute for Medical Research, P. O. Box1462, Mwanza, Tanzania

**Keywords:** Physical activity, Non-communicable diseases, Risk factors, Prevalence, Tanzania

## Abstract

**Background:**

Insufficient physical activity (PA) is a major contributing factor in the growing problem of non-communicable diseases (NCDs) in urban and rural Sub-Saharan Africa. This study aimed to determine PA and associated factors among adults in Northern Tanzania.

**Methods:**

We analyzed secondary data from a cross-sectional serological survey nested within the Magu health and demographic sentinel surveillance population in Magu District Northwestern Tanzania. All resident adults aged 15 years and older were invited to participate in the study, and physical activity data were analyzed for 5663 participants. Data were analyzed using Stata version 13.0. We used logistic regression to obtain odds ratios and 95% confidence intervals (CI) for risk factors associated with differences in PA.

**Results:**

In this mainly rural population, 96% reported sufficient PA, with a higher proportion in males (97.3%) compared to females (94.8%). In males the odds of sufficient PA were lower in rural areas compared to urban areas (OR = 0.19; *P* < 0.001; 95% CI = 0.08–0.42), while in females the odds of sufficient PA were higher in rural areas compared to urban areas (OR = 2.27; *P* < 0.001; 95%CI = 1.59–3.24). Leisure-related activity was low compared to work-related and transport-related activity. Farmers had a higher odds of sufficient PA than those in professional jobs in both males (OR = 9.75; *P* < 0.001; 95% CI = 3.68–5.82) and females (OR = 2.83; *P* = 0.021; 95% CI = 1.17–6.86).

**Conclusion:**

The prevalence of PA in this population was high. However, there is need for PA programs to maintain the high level of compliance during and following the transition to a more urban-based culture.

## Background

Globally, non-communicable diseases (NCD) account for 3.2 million deaths annually (1). Insufficient physical activity (PA) is a major contributor to NCD and is associated with a 20% increased risk of breast cancer, a 30% increased risk of cardiovascular disease, a 27% increased risk for colon cancer, and a 27% increased risk for diabetes [1]. In Tanzania, NCDs contribute significantly to the disease burden especially among adult populations [1–3]. In developing countries, the epidemiological and socio-demographic transition, accompanied by rapid urbanization, unhealthy diet, and changes in work patterns, contribute to insufficient PA [3]. Despite several initiatives aimed at promoting PA in developed and developing countries, NCDs remain a major public health problem [4, 5]. Hypertension is a major risk factor for cardiovascular disease, and improved PA is an important intervention to prevent hypertension [6].

The World Health Organization (WHO) categorizes PA by type with work-related, leisure-related and transport-related PA assessed using metabolic equivalence tables (MET) [5]. Some studies have shown that, regardless of place of residence males are more likely to engage in work-related PA than females [7–9], while other studies report that males are also more active than their females counterparts in leisure-time PA [10, 11]. However, other studies have shown no sex differences when all types of PA are considered [12].

A majority of studies comparing PA among males and females have been conducted in developed countries, which may not be relevant in Sub Sahara African countries. However, a multi-country study of 22 African countries in 2011 reported significant variation in the prevalence of sufficient PA ranging from 46.6% to 96% in adults aged 15 years and above [13]. Other studies also showed variations in PA between rural and urban settings, with people living in rural areas being more active than those living in urban areas, mainly due to the intense manual labor in farming [7, 14].

Understanding the prevalence of PA, the types of PA and associated factors for PA among adult males and females is important in planning appropriate interventions to prevent NCDs. This study aimed to determine the prevalence of PA, the types of PA and the factors associated with PA in a cross-sectional survey of a population-based cohort located 20 km from Mwanza city in North Western Tanzania. Due to the strong gender stratification of life, roles and physical activity in this population, separate analyses were undertaken for males and females.

## Methods

This study used secondary data from the Magu health and demographic sentinel surveillance (HDSS) cohort located in Kisesa ward, 20 km east of Mwanza City [15]. The total population of the HDSS cohort was approximately 34,000 people and, over 90% were from the Sukuma ethnic group, with the main economic activities being farming and trade of agricultural products such as milk, tomatoes, maize and rice [15, 16].

A cross-sectional survey was conducted between December 2013 and July, 2014, primarily to assess the HIV prevalence in the adult population. All eligible adults aged 15 years and older, and resident in the HDSS, were invited to participate in the study. A blood sample was taken to test for HIV using standard techniques in a reference laboratory, and a standard, structured questionnaire was used to collect demographic, socioeconomic, and PA data. This secondary analysis of the data included all participants who responded to the PA questions.

### PA measures

The PA questions were adapted from the WHO Global Physical Activity Questionnaire [17] with the main adaptation related to the seasonality of the work. The PA questions were divided into three sections: work-related PA, which was further divided into heavy or vigorous PA, and moderate PA, transport-related PA relating to walking or cycling, and leisure-related PA. Time spent on all PA types were summed to obtain the time spent per week doing each PA type.

Frequency, duration, and intensity for each type of PA were coded into metabolic equivalent tables (METs). One MET is defined as the energy expenditure while sitting quietly for one hour, which for the average adult is approximately 3.5 ml of oxygen/kg body weight/min with MET values obtained from the Compendium of PA by Ainsworth 1993 [18]. Using this standard, vigorous intensity work-related PA was assigned a value of 8.0METs, moderate intensity work-related PA was assigned a value of 4.0METs, transport-related PA was assigned a value of 4.0METs, and leisure-related PA was assigned a value of 4.0METs [8.9]. The total METs per week for all PA were calculated by multiplying the duration (minutes) of activity per week by its corresponding METs, and summed for each type of PA.

Sufficient METS was defined as ≥600 METS per week [17]. Respondents who had METs ≥600 were considered as having sufficient PA, otherwise they were considered as having insufficient PA.

### Statistical analyses

Statistical analyses were performed separately for each sex, using STATA Version 13 (Stata Corp. College Station, Texas, USA). Descriptive statistics were used to obtain the mean and standard deviation for normally distributed continuous variables, the median and inter-quartile range (IQR) for non-normally distributed variables, and the proportion for categorical variables. For each category of PA (work-related, leisure-related and transport-related), the median and inter-quartile range (IQR) were used to summarize the weekly MET in the population. Kruskal-Wallis test were used to compare the MET PA data between three or more groups, and the Mann–Whitney U-test for data with two groups. For the overall PA, the odds of sufficient PA (≥600 METS) was used in logistic regression analysis to obtain the odds ratio (OR) and 95% confidence interval (CI) for any comparisons. Factors associated with sufficient PA, using a *p*-value of <0.05 as significant, were included in multivariable logistic regression models to control for confounding factors.

## Results

### Socio-demographic and physical activities of the study population

More than 12,000 residents from the Magu HDSS were invited, and 9750 participated, in the survey. Of those who completed the survey, 5663 (58%) participants answered the PA questions, and were included in the analysis. Those who did not answer the PA questions had a similar ratio of males to females to those who answered the PA questions, but were significantly younger as participants still at school were not asked PA questions. The mean age of those in the analysis was 36.7 years for the 2663 males and 35.4 years for the 3600 females, with 2240 (62%) females and 1434 (69%) males residing in rural villages. Farming was the main occupation for 1543 (75%) males and 2643 (74%) females (Table 1).

**Table 1:** Socio-demographic characteristics of the participants stratified by sex (*N* = 5663)

Variables	Male (*n* = 2063)	Female (*n* = 3600)	
	n (%)	n (%)	Total
Age (Years) mean (sd) ^a^	36.7 (12.8)	35.4 (11.8)	5663
Age groups			
18–29	735 (35.6)	1346 (37.4)	2081
30–39	532 (25.8)	1065 (29.6)	1597
40–49	387 (18.8)	641 (17.8)	1028
50–59	289 (14.0)	414(11.5)	703
60+	120 (5.8)	134 (3.7)	254
Education level			
No education	396 (19.2)	1287 (35.8)	1683
Primary	1391 (67.4)	2073 (57.6)	3464
Secondary and above	276 (13.4)	240 (6.7)	516
Occupation			
Professionals	80 (3.9)	82 (2.3)	162
Farming	1543 (75.5)	2643 (74.1)	4186
Business	132 (6.5)	652 (18.3)	784
Skilled work	123 (6.0)	73 (2.1)	196
Unskilled work	164 (8.0)	119 (3.3)	283
Residence			
Urban	627 (30.4)	1358 (37.7)	1985
Rural	1434 (69.6)	2240 (62.3)	3674
Missing = 4			
Marital status			
Single/never married	547 (26.5)	273 (7.6)	820
Married	1409 (73.5)	2573 (92.4)	3982
Physical activity intensity			
Work-related vigorous activity			
No	201 (9.7)	580 (16.1)	781
Yes	1862 (90.3)	3020 (83.9)	4882
Work-related moderate activity			
No	359 (17.4)	656 (18.2)	1015
Yes	1703 (82.6))	2941 (81.8)	4644
HIV			
Negative	1877 (91.0)	3202 (88.9)	5079
Positive	185 (8.9)	397 (11.0)	582
Missing = 1			
Physical activity types			
Work-related physical activity	1962 (95.1)	3387 (94.1)	5349
Leisure-time physical activity	1739 (83.3)	1612 (44.8)	3351
Transport-related physical activity	2046 (99.2)	3506 (97.4)	5552

^a^ Mean age between sex (M vs. F)

Work-related PA was similar for males (95.1%) and females (94.1%), with a similar proportion undertaking moderate intensity work-related PA, but a higher proportion of males (90.3%) undertaking work-related vigorous intensity PA, than females (83.9%). Leisure-related PA was lower among the females (44.8%), compared to the males (88.3%). Almost all engaged in transport-related PA (99.2% of males and 97.4% of females), which included walking (Table 1).

Table 2 shows the median METs/week for the different activity types by the characteristics of the participants. For work-related PA, males had significantly higher median METS per week (median = 151.2; IQR = 108–151.2) than females (median = 115.2; IQR = 100.8–151.2) (*p*-value < 0.001). For leisure-related PA the median METs per week was significantly higher in males (median = 28.8; IQR = 14.4–50.4) than females (median = 7.2; IQR = 4.8–19.2) (*p*-value < 0.001). For transport-related PA, the median METs per week was significantly higher in males (median = 9.6; IQR = 3.6–14.4) than females (median = 4.8; IQR = 2.4–14.4) (*p*-value < 0.001). There were significant differences in the median METS per week for work-related PA across the different occupations (*p*-value < 0.001). For leisure-related and transport-related PA, there were significant differences in the median METS per week between those living in rural areas compared to those living in urban areas (*p*-value < 0.001 for each). However, for all types of PA there were no significant differences in median METs per week by age group (Table 2).

**Table 2:** Median METs/week for different types of physical activity by socio-demographic characteristics (*N* = 5663)

	Work related activity		Leisure time activity		Transport related activity	
Variables	n (%)	Median (METs/week) ^c^	n (%)	Median (METs/week) ^c^	n (%)	Median (METs/week) ^c^
	5349 (94)	151.2 (102–151.2)	3351 (59)	14.4 (7.2–50.4)	5552 (98)	6.0 (2.4–14.4)
Age						
18–29	1962 (94)	151.2 (102–151.2)	1544 (74)	16.8 (7.2–50.4)	2035 (97)	5.6 (2.4–14.4)
30–39	1493 (93)	124.8 (100.8–151.2)	969 (60)	14.4 (4.8–43.2)	1567 (98)	6.0 (2.4–14.4)
40–49	979 (95)	151.2 (103.2–151.2)	477 (46)	16.8 (7.2–50.4)	1011 (98)	7 (2.4–14.4)
50–59	672 (96)	151.2 (103.2–151.2)	266 (38)	14.4 (7.2–38.4)	690 (98)	7.2 (2.4–14.4)
60+	243 (96)	151.2 (104.151.2)	95 (37)	14.4 (7.2–33.6)	249 (98)	6 (2.4–14.4)
*P*-value ^a^		0.0422		<0.001		0.6642
Sex						
Male	1962 (95)	151.2 (108–151.2)	1739 (84)	28.8 (14.4–50.4)	2046 (99)	9.6 (3.6–28.8)
Female	3387 (94)	115.2 (100.8–151.2)	1612 (44)	7.2 (4.8–19.2)	3506 (97)	4.8 (2.4–14.4)
*P* value ^b^		<0.001		<0.001		<0.001
Education level						
No educ	1638 (97)	134.4 (103.2–151.2)	770 (45)	14.4 (4.8–50.4)	1631 (96)	7.2 (2.4–14.4)
Primary	3272 (94)	151.2 (103.2–151.2)	2221 (64)	16.8 (7.2–50.4)	3413 (98)	6 (2.4–14.4)
Secondary &above	439 (89)	120 (100.8–151.2)	360 (70)	14.4 (7.2–42)	508 (98)	4.8 (2.4–12)
*P* value ^a^		0.0017		0.0056		<0.001
Occupation						
Professional	118 (73)	108 (50.4–151.2)	89 (54)	14.4 (4.8–24)	157 (96)	6 (3.2–14.4)
Farming	4137 (98)	151.2 (103.2–151.2)	2545 (60)	16.8 (7.2–50.4)	4101 (97)	7.2 (2.4–14.4)
Business	609 (77)	108 (50.4–151.2)	363 (46)	9.6 (4.8–33.6)	770 (98)	4.8 (2.4–14.4)
Skilled work	168 (85)	116.4 (50.4–151.2)	136 (69)	15.6 (7.2–40.8)	192 (97)	4.8 (2.4–14.4)
Unskilled work	266 (93)	120 (104.4–151.2)	190 (67)	16.8 (7.2–50.4)	281 (99)	7.2 (3.6–16.8)
*P* value ^a^		<0.001		<0.001		<0.001
Residence						
Urban	1781 (89)	151.2 (100.8–151.2)	997 (50)	14.4 (7.2–31.2)	1964 (98)	4.8 (2.4–14.4)
Rural	3564 (97)	151.2 (103.2–151.2)	2352 (64)	16.8 (7.2–50.4)	3585 (97)	7.2 (2.4–19.2)
*P* value ^b^		0.0235		<0.001		<0.001
Marital status						
Single/never married	775 (94)	151.2 (100.8–151.2)	632 (77)	28.8 (12–52.8)	814 (99)	7.2 (2.4–19.2)
Married	4573 (94)	134.4 (102–151.2)	2718 (61)	14.4 (4.8–43.2)	3893 (97)	6 (2.4–14.4)
*P* value ^b^		<0.001		<0.001		<0.001

^a^ (Kruskal-Wallis test *P*-value)

^b^ Mann–Whitney U-test *P*-value

^c^ METS have been divided by 100

### Sufficient PA for males

Among males, 2007 (97.3%) had sufficient PA (MET ≥ 600 per week) (Table 3). Male farmers had 5-times higher odds of sufficient PA compared to male professionals (OR = 4.92; 95% CI = 1.95–12.36). Males participants residing in rural areas had 63% lower odds of sufficient PA compared to those residing in urban areas; (OR = 0.37; 95% CI = 0.17–0.79). However, the other socio-demographic characteristics of males such as age, level of education and marital status, were not associated with differences in the odds of sufficient PA. Multivariable logistic regression using all the factors associated with sufficient PA in the bivariate analysis, showed that only occupation and place of residence remained significantly associated with the odds of sufficient physical activity. Males who were farmers had 10-times higher odds of sufficient PA; (OR = 9.75; *P* < 0.001;95% CI = 3.68–5.82) compared to male professionals, adjusted for place of residence, and occupation. After adjusting for occupation, males who were residing in rural areas had 81% lower odds of sufficient PA (OR = 0.19; *P* < 0.001; 95% CI = 0.08–0.42) compared to those residing in urban areas.

**Table 3:** Socio-demographic factors associated with sufficient physical activity among males (*N* = 2063)

Characteristics	Total		Physical activity	
	N	n^d^ (%)	COR(95%CI)	AOR(95%CI)
Total	2063	2007 (97.3%)		
Age groups				
18–29	735	714 (97.1%)	1.0	
30–39	532	514 (96.6)	0.83 (0.44–1.59)	−
40–49	387	378 (97.7)	1.23 (0.56–2.72)	−
50–59	289	284 (98.3)	1.67 (0.62–0.62)	−
60+	120	117 (97.5)	1.14 (0.33–3.90)	−
Education level				
No education	396	381 (96.2)	1.0	
Primary	1391	1359 (97.7)	1.67 (0.89–3.11)	−
Secondary and above	276	267 (97.7)	1.16 (0.50–2.71)	−
Occupation				
Professionals	80	74 (92.5)	1.0	1.0
Farming	1543	1518 (98.4)	4.92 (1.95–12.36)	9.75 (3.68–5.82)***
Business	132	125 (94.7)	1.44 (0.46–4.47)	1.80 (0.56–5.74)
Skilled work	123	114 (92.7)	1.02 (0.35–3.00)	1.43 (0.47–4.33)
Unskilled work	164	155 (94.5)	1.39 (0.47–4.06)	2.02 (0.67–6.11)
Residence				
Urban	627	619 (98.7)	1.0	1.0
Rural	1434	1386 (96.7)	0.37 (0.17–0.79)	0.19 (0.08–0.42)***
Marital status				
Single/never married	547	532 (97.3)	1.0	
Married	1515	1474 (97.3)	1.01 (0.55–1.84)	−

*COR* Crude odds ratio; *AOR* Adjusted odds ratio

^d^Number of participants with sufficient physical activity (values may not sum to 2007 due to missing data)

***P value < 0.05 adjusted for occupation and place of residence

### Sufficient PA for females

Among females, 3411 (94.7%) had sufficient PA (MET ≥ 600 per week) (Table 4). Female farmers had four-times higher odds of sufficient PA compared to females who were professionals (OR = 3.71; 95% CI = 1.55–8.89). Females residing in rural areas had 5-times higher odds of sufficient PA compared to those residing in urban areas (OR = 4.46;95% CI = 3.23–6.17). Compared to females with no education, those with primary education had 47% lower odds of sufficient PA (OR = 0.53; 95% CI = 0.37–0.76), and those with secondary level education or above, had 70% lower odds of sufficient PA (OR = 0.30; 95% CI = 0.18–0.51). However, age and marital status were not associated with sufficient PA among females.

**Table 4:** Socio-demographic factors associated with sufficient physical activity among females (*N* = 3600)

Characteristics	Total	n^d^ (%)		Physical activity		
	N		COR(95%CI)	*P* Value	AOR(95%CI)	*P* value
Total	3600	3411 (94.7)				
Age groups						
18–29	1346	1268 (94.2)	1.0			
30–39	1065	1015 (95.3)	1.24 (0.86–1.79)	0.232		
40–49	641	608 (94.9)	1.13 (0.74–1.72)	0.558		
50–59	414	394 (95.2)	1.21 (0.73–2.00)	0.455		
60+	134	126 (94.0)	0.96 (0.45–2.05)	0.934		
Education level						
No education	1287	1245 (96.7)	1.0			
Primary	2073	1950 (94.1)	0.53 (0.37–0.76)	0.001		
Secondary	240	216 (90.0)	0.30 (0.18–0.51)	<0.001		
Occupation						
Professionals	82	76 (92.7)	1.0		1.0	
Farming	2643	2588 (97.9)	3.71 (1.55–8.89)	0.003	2.83 (1.17–6.86)***	0.021
Business	652	536 (82.2)	0.36 (0.15–0.85)	0.021	0.39 (0.16–0.92)***	0.032
Skilled work	73	68 (93.2)	1.07 (0.31–3.67)	0.910	1.04 (0.30–3.60)	0.942
Unskilled work	119	113 (95.0)	1.48 (0.46–4.78)	0.506	1.38 (0.42–4.47)	0.586
Residence						
Urban	1358	1223 (90.1)	1.0		1.0	
Rural	2240	2186 (97.6)	4.46 (3.23–6.17)		2.27 (1.59–3.24)***	< 0.001
Marital status						
Single/never married	273	258 (94.5)	1.0			
Married	3327	3153 (94.8)	1.05 (0.61–1.81)			

^d^ Number of participants with sufficient physical activity (values may not sum to 3411 due to missing data)

***P value < 0.05 adjusted for occupation and place of residence

Multivariable logistic regression using all the factors associated with sufficient PA in the bivariate analysis for females showed that only occupation and place of residence remained significantly associated with sufficient PA. Females farmers had three-time higher odds of sufficient PA (OR = 2.83; *P* = 0.021; 95% CI = 1.17–6.86) compared to professionals, adjusted for place of residence. Females residing in rural areas had two-times higher odds of sufficient PA (OR = 2.27; *P* < 0.001; 95% CI = 1.59–3.24) compared to those residing in urban areas after adjustment for occupation.

## Discussion

This study revealed that 96% of participants met WHO recommendations for sufficient PA. The high prevalence of sufficient PA could be due to the fact that the population sampled in this study were; working class, residing in a rural area, and involved in farming activities. The prevalence observed in our study is comparable with that reported in the 22 African countries that participated in the WHO Stepwise approach to chronic disease risk factor surveillance in which the overall prevalence ranged from 46.8% to 96%(13). However the prevalence of sufficient PA is higher than that reported in Nigeria [9].

The prevalence of sufficient PA was higher among males than females. This may be explained by higher participation of men in vigorous intensity activity compared to their female counterparts. This finding is contrary to that of developed countries where no difference in the prevalence of sufficient PA was found between males and females [10]. It is also contrary to the study among adult Lebanese which reported significantly higher PA among females compared to males [8]. On the other hand, differences in PA between the sexes have been reported in several studies in different parts of the world [9, 19]. Also in this study, males were more likely to participate in leisure-related PA than females, which is consistent with previous studies [10, 19]. The higher rate of male participation in leisure-related PA may be explained by the fact that women are more involved in household chores and taking care of the families while men have more free time to participate in leisure activities [20]. There are also more opportunities for men to participate in various leisure time sports such as football, running, and cycling compared with their female counterparts. There is a need of developing strategies to encourage women to engage in locally acceptable leisure PA.

This study showed a decrease in PA among women as education level increases, although this decrease in PA was not seen in males. A plausible explanation could be the confounding with occupation. Women with higher education work in offices with limited PA, while those with less education commonly engage in manual labor. This finding is in line with a previous study among women in Nigeria whereby the investigator reported that those with less than secondary education had a higher prevalence of PA [9]. Interventions to prevent NCD in Sub-Saharan Africa, and Tanzania in particular, should aim to increase PA among educated and professional people, who engage in less work-related PA, and have not taken up leisure-related PA [5].

In this study, females residing in rural areas had higher odds of sufficient PA than those residing in urban areas. This may be explained by the heavy work-related PA that rural individuals are involved in including farming and collecting wood, compared to the urban individuals. Thus urban populations, particularly women, should be targeted for PA interventions. Furthermore, our results are consistent with a previous study in Tanzania among urban and rural residents as well as a study across sub-Saharan Africa where urban dwellers had a significantly lower PA than rural residents [7, 21]. However, one of the interesting findings from this study was the lower odds of sufficient PA among males living in rural areas compared to those living in urban areas. An explanation could be that men who do not engage in farming work in the rural areas do not engage in other PAs, whereas males in urban areas have other opportunities for PA. Conversely women who do not engage in farming work have other household activities to perform.

### Study limitations and strengths

This study used a cross-sectional design which does not allow any inference to be drawn on the causal effect of PA and the factors associated with PA. The use of a self-reported measure of PA is subject to potential information bias including recall bias, and self-desirability bias [22]. There may be difficulty in the understanding of the levels of intensity for PA, for example what is seen as heavy activity by some, may be seen as moderate activity by others. Despite the limitations, this population-based study, determining the prevalence of PA and measuring the types of PA, gives an overview of the current status of PA in the study area. This will be a useful baseline for future studies to measure PA following the potential urbanization in the area.

## Conclusion

The prevalence of sufficient PA for both males and females was high (97.3% and 94.8% respectively). However, the proportion of participants that had sufficient recommended PA varied significantly by sex, occupation and place of residence.

Given the exceptionally high compliance with PA, we recommend strengthening activities that enhance PA promotion as indicated in the Tanzanian National Strategy for Non-Communicable Diseases (2016–2020) [23]. The national strategic plan states that, interventions targeting modifiable NCDs risk factors including PA, may cause a 10% relative reduction in the prevalence of diabetes from baseline and a 20% reduction in the overall mortality. Additionally, the interventions also may lead to a 25% relative reduction in the prevalence of raised blood pressure from baseline and 20% reduction in the overall mortality from cardiovascular diseases [23].

## Abbreviations

HDSS: Health and Demographic Sentinel Surveillance
HIV: Human immunodeficiency Virus
MET: Metabolic Equivalence Table
NCD: Non Communicable Diseases
PA: Physical Activity
WHO: World Health Organization
